# Association between self-management behaviour and quality of life in people with heart failure: a retrospective study

**DOI:** 10.1186/s12872-022-02535-7

**Published:** 2022-03-08

**Authors:** Eui-Young Choi, Jin-Sun Park, Deulle Min, Hye Sun Lee, Jeong-Ah Ahn

**Affiliations:** 1 Division of Cardiology, Gangnam Severance Hospital, Yonsei University College of Medicine, Seoul, Korea; 2 Department of Cardiology, Ajou University School of Medicine, Suwon, Korea; 3 Department of Nursing, College of Medicine, Wonkwang University, Iksan, Korea; 4 Biostatistics Collaboration Unit, Department of Research Affairs, Yonsei University College of Medicine, Seoul, Korea; 5 College of Nursing and Research Institute of Nursing Science, Ajou University, Worldcup-ro 164, Yeongtong-gu, Suwon, 16499 Republic of Korea

**Keywords:** Self-management, Quality of life, Heart failure, Prediction model

## Abstract

**Background:**

The purpose of this study was to investigate the variables that significantly associated with the quality of life in people with heart failure, and particularly, to identify the association between self-management behaviour and the quality of life.

**Methods:**

This retrospective study used data from heart failure outpatient clinics at two large tertiary medical centres in Seoul and Suwon, South Korea. We enrolled 119 participants who completed echocardiography and stress tests and responded to questionnaires on self-management behaviour and quality of life. We collected more data on sociodemographic and clinical characteristics and anthropometric and serum blood test results through electronic medical record review. We analysed data using multiple linear regression and the classification and regression tree (CART) method to explore the associated factors with the quality of life in participants with heart failure.

**Results:**

Participants’ mean age was 74.61 years, and women represented 52.1% of the sample. It showed that cardiac systolic function (β = 0.26, *p* = .013) and self-management behaviour (β = 0.20, *p* = .048) were two major associated factors with the quality of life in participants with heart failure in the multiple linear regression analysis. Also, cardiac systolic function and self-management behaviour were shown to be the primary determinants for the quality of life in those with heart failure in the CART analysis. Therefore, self-management behaviour of the participants with heart failure was a significant modifiable factor that can improve their quality of life.

**Conclusions:**

Healthcare providers should be aware of the importance of self-management in people with heart failure and help promote their quality of life by enhancing their self-management behaviour as own efforts to properly maintain and monitor the health status and prevent further worsening of heart failure.

## Background

Heart failure (HF) is a heterogeneous series of clinical syndromes associated with a poor prognosis, in which the body is unable to supply the proper amount of blood for metabolism due to decreased heart function [1]. According to 2013–2016 data from the National Health and Nutrition Examination Survey in the United States, the prevalence of HF continues to rise over time; it was estimated to be approximately 6.2 million, compared with an estimated 5.7 million between 2009 and 2012 [2]. This phenomenon has become a global problem with the increased aging population, and hospitalization due to HF is the leading cause of overall hospitalization in the United States and European countries [3, 4]. HF cannot be completely cured and requires lifelong management. Repeated hospitalizations for HF affect the health care system, resulting in a high social and economic burden [3]. A systematic review of 16 studies (between 2004 and 2016) analysed the cost associated with HF and reported that the annual medical expenses ranged from $868 to $25,532, with the lifetime cost for a person with HF estimated at $126,819 [5].

People with HF can be divided into four classes using the New York Heart Association (NYHA) classification based on the severity of symptoms and related physical effort needed [6]. They can also be divided into stages A (high risk of developing HF in the future) to D (advanced HF) [7]. The assessment for HF classification should consider not only a careful clinical evaluation but also the individual’s psychosocial factors, for instance, the quality of life (QoL), which can be a more important factor outside the hospital management [8]. People with HF usually suffer from a variety of physical symptoms such as dyspnea, dizziness, edema, lack of energy, and sleep disturbance, and psychological problems such as stress, anxiety, and depression along with changes in heart function, further reducing overall QoL [9]. The treatment goal for HF is to control the worsening symptoms, reduce re-hospitalizations, and maintain survival [10]. Accordingly, individual self-management plays an important role in HF management. Self-management is defined as a process of maintaining health status through individual’s health promoting and preventive practices [11]. For people with HF, self-management includes three key aspects of maintenance (i.e., taking medication as prescribed, engaging in physical activity, and adhering to a therapeutic diet), monitoring (i.e., regular weighing to check for body fluid accumulation and watching for symptoms of worsening), and management (i.e., controlling water or salt intake and changing medication dose in response to symptoms) [10]. People with HF need to recognise their exacerbating signs and manage related factors, and through this, they will be able to improve their QoL and lower their mortality. Thus, self-management is a necessary focus in life-long HF care, which people with HF should continue throughout their lives [10, 11], while healthcare providers should ensure the best possible QoL in those with HF [12].

Recently, many studies on self-management and QoL in people with HF have been conducted. However, according to a systematic review of 30 studies, there was a discrepancy among the individual study results, which examined the relationship between health-related QoL and self-management in those with HF [13]. The discrepancy also appeared in interventional studies. One systematic review of 19 randomised controlled trials reported that some self-management interventions significantly affected the QoL of participants with HF, but others did not [14]. As such, many studies have emphasised the importance of self-management and QoL in people with HF; however, their results have been inconsistent. The purpose of this study was to consider various possible factors associated with the QoL in participants with HF, and particularly, to investigate the association between self-management behaviour and the QoL.

## Methods

### Study design and participants

This study used a retrospective observational design. Participants for the present study were adults with HF who visited the outpatient departments of two large tertiary medical centres operating HF outpatient clinics in two cities of metropolitan area (Seoul and Suwon city), South Korea, for regular medical follow-ups between July 2017 and August 2019. We selected 119 participants who had performed relevant serum blood tests, echocardiography, and stress tests and responded to the surveys about self-management behaviour and the QoL on the same day. We collected their data retrospectively by electronic medical record review.

### Study variables

Self-management behaviour was measured using the European Heart Failure Scale [15], a 12-item questionnaire related to self-care behaviour in HF. It includes the questions of consulting behaviours (i.e., “How often do you call your doctor/nurse in case of shortness of breath, ankle swelling, weight gain, or fatigue?”) and adherence with the regimen (i.e., “How often do you weigh yourself, try to drink less water, follow a low-sodium diet, regularly take medication, or exercise?”). Also, their QoL was assessed using a measuring tool provided by the World Health Organization (WHOQOL-BREF) [16], a 26-item questionnaire on the individual’s perceptions of their health and well-being. The participants’ stress levels were measured using the heart rate variability (HRV) measurement tool of uBioMacpa (Biosense Creative, Seoul, Korea), which displays stress level on a scale of 0 to 100.

All participants underwent a comprehensive transthoracic echocardiographic evaluation, a standard 2-dimensional and Doppler echocardiographic examination, according to the recommendations of the American Society of Echocardiography [17]. Left ventricular systolic function was defined using the left ventricular ejection fraction (EF), calculated according to the modified Simpson’s method (i.e., subtracting left ventricular end-systolic dimension from left ventricular end-diastolic dimension). Left ventricular diastolic function was defined as the early mitral inflow velocity to early diastolic mitral septal annular velocity (E/E’), calculated using pulsed-wave Doppler and tissue Doppler echocardiography. The evaluation was conducted using GE Vivid 7 (GE Healthcare, Horten, Norway) or iE33 (Philips Medical Systems, Andover, MA, USA), performed by 6 sonographers and 2 echocardiologists in one medical centre. In the other medical centre, it was conducted using Vivid E95 (GE Healthcare, Horten, Norway) or EPIQ CVX (Philips Medical Systems, Andover, MA, USA), which was performed by 8 sonographers and 2 echocardiologists. In this study, we only collected EF for cardiac systolic function and E/E’ for cardiac diastolic function from the participants’ echocardiographic results.

Electronic medical record review was performed to collect the participants’ sociodemographic and clinical characteristics, anthropometric data, and serum blood test results, including hemoglobin A1C (HbA1C), high-density lipoprotein (HDL), low-density lipoprotein (LDL), total cholesterol, triglyceride, and high sensitivity C-reactive protein (hs-CRP).

### Statistical analyses

Data were analysed using SPSS version 25.0 (IBM Corporation, Armonk, NY, USA).

Descriptive statistics (frequencies, percentages, means, and standard deviations) were used to explain the participants’ sociodemographic and clinical characteristics, levels of stress, self-management behaviour, and QoL. Independent samples *t*-tests and χ^2^ tests were conducted to identify the differences in the variables according to the levels of low and high QoL. The two QoL levels were created by using a median split for the QoL measure.

Multiple linear regression analysis was performed to examine the relationships among QoL, EF, E/E’, and self-management behaviour. The choice of these variables for the regression analysis was based on the significance in the univariate analysis to identify the major factors that predict the QoL.

Lastly, the predictive model for QoL of participants was developed using decision tree analysis. Decision tree analysis is a data-mining technique designed to partition the whole dataset into subgroups based on splitting criteria [18]. We used the classification and regression tree (CART) method [18], which presents a hierarchical model structured as a tree for predicting the QoL of the participants. The tree model structure is made up of root nodes, splitting nodes (parent nodes), and terminal nodes (child nodes). Firstly, the dataset was partitioned into two subsets based on a predictor variable with the score of QoL. The process was repeated on each derived subset in an iterative (recursive partitioning) manner. This method looks for subgroups in the dataset in which the predictor variable is relatively homogeneous. At each node, the recursive partitioning identifies a predictor variable and a split by which may be subclassified [19].

## Results

The mean age of the participants was 74.61 years, and 52.1% were women. The differences in the variables according to the groups with low and high QoL are presented in Table 1. There were statistically significant differences in EF (*p* < 0.001), E/E’ (*p* = 0.045), and self-management behaviour (*p* < 0.022) between low and high QoL groups. Participants with high QoL showed significantly higher EF, lower E/E’, and better self-management behaviour scores than those with low QoL. No other statistically significant differences between the groups were observed.

**Table 1:** Participants’ sociodemographic and clinical characteristics (*N* = 119)

Characteristics	Low QoL (*n* = 59)	High QoL (*n* = 60)	*p*
	n (%) or mean ± SD		
Age (range: 35–96)	74.98 ± 10.87	74.23 ± 11.75	.719
< 60	4 (6.8)	7 (11.7)	.651
60–69	13 (22.0)	9 (15.0)	
70–79	19 (32.2)	21 (35.0)	
≥ 80	23 (39.0)	23 (38.3)	
Spouse*			.476
Yes	35 (60.3)	40 (66.7)	
No	23 (39.7)	20 (33.3)	
Educational level*			.492
≤ Middle school	32 (56.1)	33 (55.0)	
≤ High school	17 (29.8)	14 (23.3)	
≥ College/university	8 (14.0)	13 (21.7)	
Occupation*			.562
Yes	11 (19.0)	14 (23.3)	
No	47 (81.0)	46 (76.7)	
Family history*			.416
Yes	9 (15.8)	13 (21.7)	
No	48 (84.2)	47 (78.3)	
Body mass index (kg/m^2^)	24.45 ± 4.29	24.69 ± 3.11	.733
Waist circumference (cm)	88.54 ± 10.10	88.29 ± 10.06	.919
Heart failure duration (y)	7.23 ± 4.87	8.62 ± 5.57	.155
Number of hospitalization	1.28 ± 0.97	1.08 ± 0.88	.286
Treatment*			
Medication			.981
Yes	57 (98.3)	59 (98.3)	
No	1 (1.7)	1 (1.7)	
Internal Intervention			.115
Yes	19 (32.8)	12 (20.0)	
No	39 (67.2)	48 (80.0)	
Surgery			.411
Yes	10 (17.2)	14 (23.3)	
No	48 (82.8)	46 (76.7)	
NYHA class*			.222
I	9 (17.6)	15 (25.4)	
II	23 (45.1)	30 (50.8)	
III	15 (29.4)	8 (13.6)	
IV	4 (7.8)	6 (10.2)	
Systolic blood pressure (mmHg)	121.51 ± 17.77	127.33 ± 14.37	.501
Diastolic blood pressure (mmHg)	68.93 ± 11.84	73.07 ± 13.52	.079
HbA1c (%)	6.64 ± 1.10	6.88 ± 1.10	.517
HDL (mg/dL)	49.47 ± 15.06	46.77 ± 11.95	.319
LDL (mg/dL)	85.76 ± 37.36	85.13 ± 30.38	.926
Total cholesterol (mg/dL)	147.00 ± 47.50	150.81 ± 34.25	.619
Triglyceride (mg/dL)	115.86 ± 65.05	133.43 ± 71.18	.195
hs-CRP (mg/dL)	1.15 ± 1.45	2.88 ± 6.02	.215
EF (%)	50.17 ± 19.01	60.92 ± 13.27	< .001
E/E’	16.93 ± 8.69	14.03 ± 5.97	.045
Stress (0–100)	50.23 ± 20.45	40.33 ± 21.93	.203
Self-management behaviour (1–5)	3.28 ± 0.60	3.54 ± 0.56	.022

QoL, quality of life; NYHA, New York Heart Association; HbA1C, hemoglobin A1c; HDL, high density lipoprotein; LDL, low density lipoprotein; hs-CRP, high sensitive C-reactive protein; EF, ejection fraction; E/E′, early mitral inflow velocity/early diastolic mitral annular velocity

*Excluded, no response

The factors that significantly associated with the participants’ QoL are shown in Table 2. Multiple linear regression analysis was performed with EF, E/E’, and self-management behaviour as the independent variables based on their significance in the univariate analysis to identify the major factors that predict the QoL. The regression model for the participants’ QoL was shown to be significant (*p* = 0.003). The value of the adjusted *R*^2^ was 0.11, corresponding to the explanatory power of 11.0% for QoL. The major factors associated with the QoL were EF (β = 0.26, *p* = 0.013) and self-management behaviour (β = 0.20, *p* = 0.048).

**Table 2:** Factors associated with quality of life in participants with heart failure

Variables	*B*	SE (*B*)	β	*p*
EF	0.01	0.01	0.26	.013
E/E′	− 0.01	0.01	− 0.04	.665
Self-management behaviour	0.23	0.12	0.20	.048
Overall: *R*^2^ = .14, Adjusted *R*^2^ = .11, *F* = 5.03, *p* < .003				

EF, ejection fraction; E/E′, early mitral inflow velocity/early diastolic mitral annular velocity

**Fig. 1:**
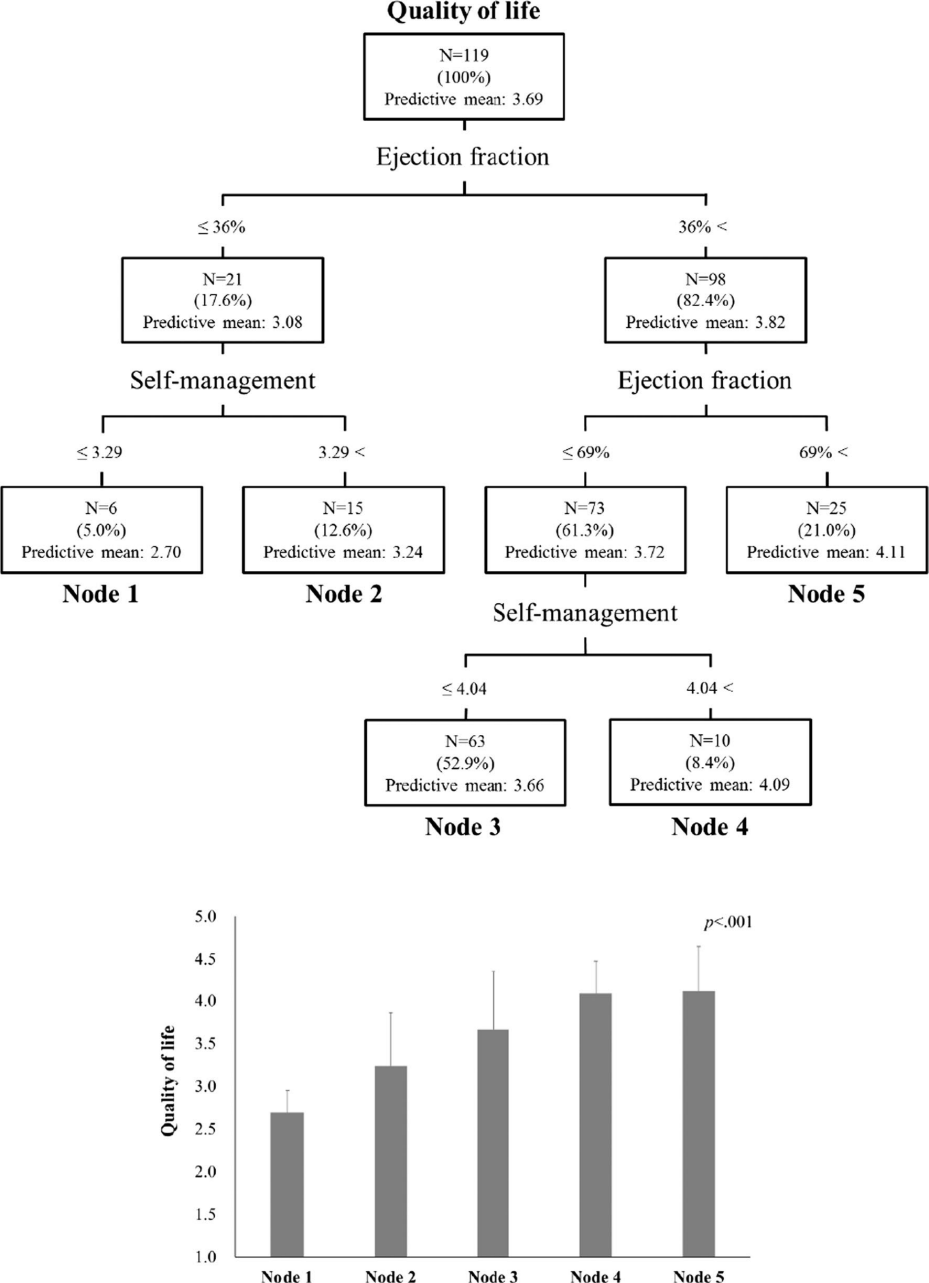
Classification and regression tree for quality of life in participants with heart failure

To perform the CART analysis, we selected EF and self-management behaviour as the candidate predictors based on the regression analysis. The prediction model by CART analysis for the participants’ QoL is shown in Table 3 and Fig. 1. The EF (cut-off value: 36%) was shown to be the primary determinant of the participants’ QoL. The lowest QoL group (Node 1; predictive QoL value of 3.08 out of 5) with 6 participants (5.0%) had EF ≤ 36%, and their self-management score was lower than 3.29 out of 5. Contrarily, the highest QoL group (Node 5; predictive QoL value of 4.02) with 25 participants (21.0%) had EF > 69%. In the group with EF ≤ 36%, if the participants’ self-management score was higher than 3.29 (15 participants, 12.6%), they showed a predictive QoL value of 3.24 (Node 2). The group, which had EF between 37 and 69%, was divided into two nodes (Nodes 3 and 4). Node 3 (predictive QoL value of 3.66) included participants with self-management behaviour score ≤ 4.04 (63 participants, 52.9%), and Node 4 (predictive QoL value of 4.09) included participants with self-management behaviour score > 4.04 (10 participants, 8.4%).

**Table 3:** Quality of life in participants with heart failure of each node based on CART

Node	Definition	n (%)	mean ± SD	*B*	SE (*B*)	β	*p*
Node 1	EF ≤ 36 & Self-Management ≤ 3.29	6 (5.0)	2.70 ± 0.25				
Node 2	EF ≤ 36 & Self-management > 3.29	15 (12.6)	3.24 ± 0.62	0.54	0.30	0.26	.043
Node 3	36 < EF ≤ 69 & Self-Management ≤ 4.04	63 (52.9)	3.66 ± 0.69	0.97	0.27	0.69	< .001
Node 4	36 < EF ≤ 69 & Self-Management > 4.04	10 (8.4)	4.09 ± 0.39	1.39	0.32	0.55	< .001
Node 5	EF > 69	25 (21.0)	4.11 ± 0.54	1.42	0.28	0.82	< .001
Overall: *R*^2^ = .26, Adjusted *R*^2^ = .23, *F* = 9.80, *p* < .001							

CART, classification and regression tree; EF, ejection fraction

## Discussion

This study attempted to explore the factors associated with the QoL in people with HF and the importance of self-management on their QoL. Among the participants’ various physical, psychological, behavioural, and diagnostic test results, EF and self-management behaviour were factors that significantly associated with their QoL.

Previous studies have shown that EF is an important hallmark in people with HF that reflects the disease prognosis and outcomes, such as worsening symptoms, hospital readmission, mortality, and QoL [9, 20, 21]. Since HF cannot be ultimately cured, a necessary treatment strategy is to maintain the functional capacity and improve the QoL by continuous lifetime monitoring with the cooperation of healthcare providers and the individuals themselves [10, 22]. Regular observation of the echocardiography results is essential to manage treatment goals in people with HF, as it is a simple and intuitive measurement for the evaluation of EF. Although increased EF can bring satisfaction to healthcare providers and people with HF, it is not easy to improve. Various medical treatments, such as pharmacological therapy, cardiac revascularization, resynchronization, and ventricular assist devices, have been availed of to improve the EF in people with HF; however, everyone does not get complete improvement with uniform treatment, so various studies are ongoing to determine the most favourable and optimal treatment [23, 24]. In addition, measuring EF through echocardiography has also been reported to have limitations, such as limited reliability due to inter- and intra-observer variability and poor image quality [25, 26]. Further, the concerns that QoL and the diverse symptoms of HF are not always associated with EF, which is a useful but simplistic parameter to assess the complexity of HF, should be considered in clinical practice [27].

Self-management behaviour can be a modifiable factor in improving QoL in people with HF. In the present study, self-management of the participants was one of the significant factors associated with their QoL. As we further noticed with the prediction model using the CART analysis that identified significant predictor variables and the splits, even in the low EF group, if the self-management behaviour score was relatively high, the relative QoL score was also high. It is in line with the results of a recent systematic review that showed evidence that people with HF can improve their QoL by promoting self-care behaviours [13]. Previous studies suggested that self-management interventions like education, support, and guidance can improve the QoL in people with HF using diverse delivery methods such as face-to-face interaction, telephonic conversation, accessing websites, mobile applications [28–31].

Self-management of HF is the individual’s comprehensive behaviour, including maintaining self-care for physical and psychological stability and self-monitoring the possible worsening signs and symptoms [10]. Maintaining self-care includes taking prescribed medications, doing proper and regular physical activity, limiting salt and water uptake, keeping an adequate body weight, and so on. Self-monitoring also includes observing the signs and symptoms related to HF experienced by themselves and responding appropriately before advanced outcomes occur [10, 32]. For people with chronic conditions like HF, self-management represents a critical strategy for improved treatment outcomes that they should accept as an aspect of daily routine for their lifetime rather than a short-term event [33]. Nevertheless, it is an ongoing challenge for healthcare providers and people with HF to enable self-management behaviour and continue to be stable without giving up. Some studies emphasised the role of people with HF in decision-making based on the knowledge and trial and error experience for self-management adherence [34–36]. Additionally, some studies highlighted the role of healthcare providers in improving self-management in people with HF through constant and multifaceted efforts, such as interactive education, teach-back, retraining, and support using diverse and customised delivery methods [28, 29, 37]. Regardless of the initial low or high EF, efforts to improve the self-management ability of people with HF will both promote their self-care and ultimately contribute to the achievement of the goal of treatment by enhancing the QoL.

This study has several limitations. First, this was a retrospective study based on a relatively small and convenient sample from two large tertiary medical centres in South Korea, and missing data existed in some variables, which may not represent the population and therefore has poor generalisability. Second, there may be differences in application to other participants since we analysed using the median value of the QoL. Third, we used the E/E’ as a representative value for cardiac diastolic function in this study. However, diverse parameters, such as left atrial volume index, lateral early diastolic mitral annular velocity, the ratio of early diastolic transmitral flow velocity to late diastolic transmitral flow velocity (E/A), and E-wave deceleration time, can be considered for assessing diastolic function, and the assessment method we used is not applicable to certain populations with arrhythmia, mitral stenosis, mitral regurgitation, or mitral valve prosthesis [38]. In addition to the quantitative variables of EF and E/E’, the qualitative variables of left ventricular systolic dysfunction and diastolic dysfunction should be considered. Lastly, we performed regression analyses with independent variables based on the significance only in the univariate analysis; however, sociodemographic characteristics should have been considered which may be confounding variables on the associations in the study. Future research should be expanded to include an increased number of participants and comprehensive variables and both quantitative and qualitative measurement tools to examine the validity of the prediction model using CART analysis presented in this study. Nevertheless, this study has strength in confirming that self-management is an important factor associated with the QoL in people with HF.

## Conclusions

The EF and self-management behaviour are factors significantly associated with the QoL in people with HF. Furthermore, self-management behaviour should be considered as an important and modifiable factor that can increase QoL as a treatment goal of people with HF. Further ongoing research is needed to understand ways of effectively improving self-management adherence in people with HF.

## Data Availability

The data that support the findings of this study are available from the authors upon reasonable request and with permission of the medical centres where the authors collected the data retrospectively.

## Abbreviations

CART: Classification and regression tree
E/A: Early diastolic transmitral flow velocity to late diastolic transmitral flow velocity
E/E′: Early mitral inflow velocity to early diastolic mitral septal annular velocity
EF: Ejection fraction
HbA1C: Hemoglobin A1C
HDL: High-density lipoprotein
HF: Heart failure
HRV: Heart rate variability
hs-CRP: High sensitivity C-reactive protein
LDL: Low-density lipoprotein
NYHA: New York Heart Association
QoL: Quality of life
WHOQOL-BREF: World Health Organization quality of life instrument short form
